# The influence of leader humor on employee creativity: from the perspective of employee voice

**DOI:** 10.3389/fpsyg.2023.1162790

**Published:** 2023-06-05

**Authors:** Yi Cao, Keqiucheng Zhou, Yijiang Wang, Yubo Hou, Rentao Miao

**Affiliations:** ^1^School of Psychological and Cognitive Sciences, Beijing Key Laboratory of Behavior and Mental Health, Peking University, Beijing, China; ^2^Renmin University of China, Beijing, China; ^3^Capital University of Economics and Business, Beijing, China

**Keywords:** contradiction thinking, creativity, employee voice, leader humor, ownership

## Abstract

Creativity is a primary factor in driving transformational change. This study explored the impact of leader humor on employee creativity (incremental and radical creativity) from the perspective of employee voice. Data were collected from 812 Chinese employees through multipoint surveys. Through the surveys, we found that (1) leader humor has a significant positive effect on employee incremental and radical creativity; (2) promotive/prohibitive voice mediates the relationship between leader humor and employee incremental/radical creativity separately; (3) contradictory thinking moderates the effect of leader humor on prohibitive voice and further moderates the indirect effect of leader humor on radical creativity; and (4) the moderated mediation model mainly applies to state-owned enterprises rather than private-owned enterprises. The theoretical and practical implications of these findings are discussed.

## Introduction

In recent years, industries have struggled in economically uncertain times, especially due to the COVID pandemic and trade war between China and the United States, and some enterprises are on the verge of bankruptcy. However, some enterprises driven by creativity have shown strong adaptability and even achieved tremendous development during this period. This is because creativity is the key to the survival and development of enterprises (Hughes et al., 2018). Employee creativity is the micro-foundation of enterprise creativity (Liu et al., 2017) and is the key element leading to innovation (Gilson and Madjar, 2011). Thus, stimulating employee creativity in uncertain environments has become a priority for firms and leaders.

A substantial body of literature has explored the origins of employee creativity from the perspective of leadership and found some important results (Wang and Rode, 2010; Zhang and Bartol, 2010; Rego et al., 2012; Qu et al., 2015; Byun et al., 2016; Chen and Hou, 2016; Ma and Jiang, 2018; Cai et al., 2019; Koh et al., 2019; Younas et al., 2020; Liang et al., 2021). However, humor, as an important component of successful leadership (Yam et al., 2018) with the ability to produce a range of positive outcomes in the workplace (Karakowsky et al., 2020), has not received enough attention. Recent research has found that employees prefer humorous leaders over ethical leaders (Yam et al., 2019) since humorous leaders always motivate their subordinates and create good, lasting memories (Cooper et al., 2018). Given that it is important to explore the impact of leader humor on employee creativity, some studies have been conducted in this area of research (Lee, 2015; Li et al., 2019; Hu, 2020; Peng et al., 2020; Yang and Yang, 2021). These studies, however, have only regarded creativity as a single concept, which overlooks its inherent complexity, causing the effect and the mechanism of leader humor influencing employee creativity to be ambiguous. In previous studies, creativity was conceptualized and operationalized as a unidimensional construct, often defined as the creation of new and applicable ideas (Amabile et al., 1996; Zhou and George, 2001). However, recent studies have increasingly discussed distinct types of creativity, ranging from minor adaptations to radical breakthroughs (Malik and Choi, 2019). Madjar et al. (2011) characterized creativity as incremental and radical. Incremental creativity refers to small changes and modifications to existing practices in the organization, focusing on the exploitation of ideas, whereas radical creativity involves new ideas that are completely different from the existing practices of the organization, emphasizing the exploration of ideas (Litchfield, 2008; Madjar et al., 2011). Recently, the literature has acknowledged the distinction between radical and incremental creativity (Xu and Jiang, 2018; Malik and Choi, 2019; Acemoglu and Akcigit, 2022). Both types of creativity are key drivers of organizational performance and are equally important for organizational development (Gilson and Madjar, 2011). Nonetheless, previous studies did not investigate or further distinguish the mechanism by which leader humor influences these two types of creativity.

The present study proposes that the mechanism between leader humor and the two types of creativity can be explained from the perspective of employee voice. Employee voice is an organizational citizenship behavior that is both positive and challenging (Lepine and Van Dyne, 1998). This behavior involves questioning and challenging the current state of the organization and even jeopardizing the employee's relationship with leaders with certain interpersonal risks (Liu and Zhu, 2010). However, according to the Benign Violation Theory (BVT), leader humor may promote employee voice (BVT; McGraw, 2010). BVT indicates that the display of humor often necessitates a benign norm violation (Yam et al., 2018). In other words, it explains how humor turns threatening or challenging violations into benign violations (Cheng et al., 2021). In light of this, leader humor may reduce the risk of employee voice and gives employees the courage to express constructive opinions on issues.

Furthermore, although some studies have highlighted a significant and positive association between voice behavior and creativity (Ng, 2012; Soomro and Memon, 2020), the type of creativity exhibited may vary with employee voice. Liang et al. (2012) classified employee voice into promotive and prohibitive voices; the former comprises employees' expressions of new ideas or suggestions for *improving* the overall functioning of the organization, and the latter comprises employees' expressions of concern about work practices that are harmful to *reforming* the overall functioning of the organization. Therefore, although the two types of voices both challenge the status quo of the organization, they have different functions and purposes (Liang et al., 2012), which may be associated with different types of creativity.

In addition, according to the incongruity theory, humor emerges when certain concepts or ideas are inconsistent with their true expressions (Attardo, 1997). As a result, the emergence of humor often involves a contradiction, and thus, individuals' contradictory thinking may become the premise of experiencing humor. Therefore, although leader humor may promote employee voice, the relationship also depends on employees' contradictory thinking. Moreover, corporate ownership is the major institutional factor in China (Liu et al., 2017), as employee treatment varies with ownership. While private-owned enterprises (POEs) are more market-oriented and have more open work atmospheres, which encourage employees to provide suggestions or develop new ideas, state-owned enterprises (SOEs) have clear hierarchical boundaries, stricter divisions of labor, and a more serious work atmosphere, which is not conducive to employee voice. Thus, ownership may further interact with employees' contradictory thinking to moderate the impact of leadership humor on employee voice.

In summary, the present study examined the influence of leader humor on different types of employee creativity and how the effects may be mediated by types of employee voice and moderated by employees' contradictory thinking and corporate ownership.

## Hypothesis development

### Leader humor and employee creativity

Leader humor is social behavior performed by leaders to delight employees (Cooper et al., 2018). Substantial studies have found that leaders with a sense of humor produce a series of positive results on employees' attitudes and behaviors, such as obtaining greater support from them (Mao et al., 2017), minimizing their withdrawal behaviors (Mesmer-magnus et al., 2012), promoting their organizational citizenship behaviors (Cooper et al., 2018), and enhancing their perceived wellbeing, work commitment, and innovation (Arendt, 2006; Ünal, 2014). In light of previous research, the current study proposes that leader humor promotes employee creativity.

First, according to the broaden-and-build theory, compared to negative affect, positive affect broadens the momentary thought-action repertoire and builds lasting personal resources, which prompts individuals to discard time-tested or automatic behavioral scripts and to pursue novel, creative, and often unscripted paths of thought and action (Fredrickson, 1998). In this respect, the expression of positive change through leader humor may stimulate creativity. Second, humorous leaders have the ability to foster a sense of closeness among employees, eliminating hierarchical differences between them (Romero and Cruthirds, 2006; Kim et al., 2016) and creating a more open communication environment (Mao et al., 2017). It thus provides the foundation for employees to express their creative ideas freely. Third, leader humor conveys the leader's trust and support for employees, which helps form a high-quality leader–subordinate relationship. Based on the principle of reciprocity, to maintain this relationship, employees are more likely to work hard to improve or change their workflow in innovative ways (Zhang and Su, 2020). Therefore, we propose the following:

*H1a:* Leader humor is positively correlated with incremental creativity.
*H1b:* Leader humor is positively correlated with radical creativity.

### The mediating role of employee voice

Based on the BVT (McGraw, 2010), humor involves violations of norms (Yam et al., 2018), which tells people how to evaluate the challenging contexts they face, i.e., the extent to which they perceive them as relatively benign rather than threatening. Cheng et al. (2019, 2021) demonstrated that humor may give individuals a sense of control in challenging situations (e.g., stressful situations), which can help them manage challenges. Although voice behavior is challenging for employees in the workplace, leader humor signals to employees that it is socially acceptable. Therefore, leader humor may reduce the risk of employee voice and give employees the courage to express constructive opinions on issues.

Although the two types of voices challenge the status quo and are aimed at benefiting organizations (Liang et al., 2012), they may lead to different levels of creativity regarding the targets of promotive (e.g., improving the organization) and prohibitive (e.g., reforming the organization) voices. A promotive voice is generally considered more broadly applicable and is easily acceptable by organizations and leaders (Morrison, 2014) because it is ultimately expected to benefit the whole organization (Liang et al., 2012). However, a prohibitive voice is generally riskier and more challenging, thereby inducing conflict and negative emotions among coworkers and supervisors (Liang et al., 2012). As a result, a prohibitive voice is likely to generate resistance from organizations and leaders (Burris, 2012).

In this respect, a promotive voice represents small changes and modifications to existing practices and thus tends to be more practical and exploitative. Moreover, compared to prohibitive voice, promotive voice reflects employees' recognition of the existing system. Therefore, a promotive voice aims to continuously improve and exploit the existing system rather than make explorative changes (Lin and Johnson, 2015). A prohibitive voice is associated with the potential problems of the organization (Miao et al., 2020) and requires employees to “think outside the box.” As a result, a prohibitive voice is explorative and often opposed to the organization's existing system (Morrison, 2014).

Moreover, employees who propose a prohibitive voice tend to take risks or challenge the status of the organization, and thus, they are not bound by pressure from leaders. The absence of external pressure results in no or low restrictions on the creative behavior these employees exhibit. Exploitation conforms to the characteristics of incremental creativity, while exploration combines the characteristics of radical creativity (March, 1991; Benner, 2003). Given that, we propose the following:

*H2a:* A promotive voice mediates the relationship between leader humor and incremental creativity.
*H2b:* A prohibitive voice mediates the relationship between leader humor and radical creativity.

### The moderating effect of contradictory thinking

The incongruity theory claims that humor is often related to inconsistency (Attardo, 1997), which shows that humor is often accompanied by contradiction. People with high levels of contradictory thinking believe that contradiction is a natural, inherent, and inevitable feature of virtually all existence (Spencer-Rodgers, 2017), and thus, they are more likely to be aware of inconsistencies in the surrounding environment. In contrast, people with low levels of contradictory thinking are less sensitive to inconsistency. As a result, not all employees can perceive the humor expressed by leaders. Only employees with high levels of contradictory thinking can perceive the inconsistency embedded in humorous language and behaviors and thus better understand leader humor. Given that, we propose the following:

*H3a:* Contradictory thinking moderates the relationship between leader humor and promotive voice; leader humor has a stronger effect on the promotive voice of employees with high levels of contradictory thinking than on the promotive voice of employees with low levels of contradictory thinking.
*H3b:* Contradictory thinking moderates the relationship between leader humor and prohibitive voice; leader humor has a stronger effect on the prohibitive voice of employees with high levels of contradictory thinking on the prohibitive voice of employees with low levels of contradictory thinking.

Moreover, this moderating model may be further influenced by the mediating role of employee voice. Specifically, employee voice mediates the relationship between leader humor and employee creativity; however, the effect size of this mediation depends on employees' contradictory thinking. Since leader humor has a strong impact on the voice behavior of employees with high levels of contradictory thinking, the mediating effect of employee voice on the relationship between leadership humor and creativity is expected to be stronger. In contrast, when employees have low levels of contradictory thinking, leadership humor has a weak influence on employee voice, and thus, the mediating effect of employee voice in the relationship between leadership humor and employee creativity is expected to be weaker.

*H4a:* Contradictory thinking has a moderating effect on the mediating role of promotive voice in the relationship between leader humor and incremental creativity (*H2a*). Leader humor has a stronger indirect effect on the incremental creativity of employees with high contradictory thinking than on the incremental creativity of employees with low contradictory thinking.
*H4b:* Contradictory thinking has a moderating effect on the mediating role of a prohibitive voice in the relationship between leader humor and radical creativity (*H2b*). Leader humor has a stronger indirect effect on the radical creativity of employees with high levels of contradictory thinking than the radical creativity of employees with low levels of contradictory thinking.

### The moderating role of corporate ownership

Based on H4a and H4b, the present study further proposes that the moderated mediation effect of contradictory thinking may also be influenced by corporate ownership. Ownership is a common characteristic of Chinese enterprises (Peng and Luo, 2000). Although Chinese SOEs are market-oriented, they still retain traditional management styles. For example, many SOEs still adopt a bureaucratic management system with hierarchical and centralized characteristics, which is not conducive to encouraging employee voice. In contrast, POEs tend to be highly market-oriented and have a flatter management structure and a freer working atmosphere, thus encouraging employee voice. Recently, some POEs have established “suggestion boards” on their intranet systems to encourage employees to make suggestions for improving the enterprise. Thus, hypotheses 4a and 4b may not be supported in the case of SOEs.

*H5a:* Ownership moderates *H4a*; the moderating effect of contradictory thinking on the mediating role of a promotive voice in the relationship between leader humor and incremental creativity is stronger in POEs than in SOEs.
*H5b:* Ownership moderates *H4b*; the moderating effect of contradictory thinking on the mediating role of a prohibitive voice in the relationship between leader humor and radical creativity is stronger in POEs than in SOEs.

**Figure 1:**
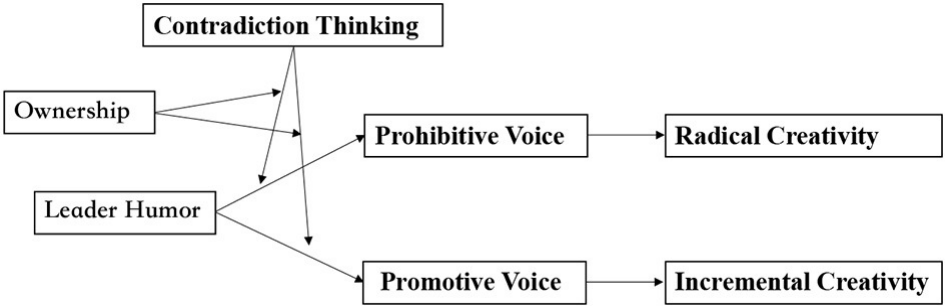
Research framework.

The research framework is illustrated in Figure 1.

## Method

### Participants and procedure

A total of 1,000 full-time employees were invited to participate in the survey. The participants did not know the research framework; they were only informed that the data would be used for statistical analysis rather than for other purposes. In addition, the participants included in the sample needed to have daily interactions with their direct supervisors. All data were collected at three time points. At time point 1, 1,000 participants rated their leader's humor, as well as their contradictory thinking and demographic information, and 937 valid questionnaires were collected. At time point 2, 937 participants rated their promotive and prohibitive voices in their workplace, and 881 valid questionnaires were collected. At time point 3, 881 participants were asked to rate their radical and incremental creativity, and 832 valid questionnaires were collected. After excluding unqualified samples (from those who failed to pass the quality check questions), 812 valid questionnaires were obtained.

Among the participants, 427 (52.6%) of them were from SOEs, including 150 men (35.1%) and 277 women (64.9%), with an average age of 31.37 years (SD = 8.319); 164 (38.4%) of them were unmarried, and 263 (61.6%) were married; 103 (24.1%) of participants had a senior high school or lower level of education, 124 (29.0%) had a junior college education, and 200 (46.9%) had an undergraduate or higher level of education. The average number of years they had worked in the current firm was 7.03 years (SD = 7.923). Moreover, 385 (47.4%) participants were from POEs, including 201 men (52.2%) and 184 women (47.8%), with an average age of 32.58 years (SD = 7.722); 144 (37.4%) of these participants were unmarried and 241 (62.6%) of them were married; 45 (11.7%) of them had a senior high school or lower level of education, 59 (15.3%) had a junior college education, 281 (73%) had an undergraduate or higher level of education; and the average years they worked in their current firm was 4.79 years (SD = 5.413).

### Measures

All items in the questionnaire were rated on a 7-point Likert scale, ranging from 1 (completely disagree) to 7 (completely agree).

#### Leader humor

The Leader Humor Scale developed by Cooper et al. (2018) was used to measure leader humor. The scale contains three items, including: “In many situations, my leader will use humor to communicate with me.” The internal consistency coefficient (α), the McDonald's ω, the composite reliability (CR), and the average variance extracted (AVE) of the scale were 0.921, 0921, 0.950, and 0.863, respectively.

#### Employee voice

The scale proposed by Liang et al. (2012) was adopted to measure employee voice. The scale contains two subscales that measure a promotive and a prohibitive voice. The subscale of promotive voice contains five items, including “I proactively suggest new projects that are beneficial to the work unit,” while the subscale of prohibitive voice contains six items, including “I am willing to point out problems when they occur, even if it could hamper my relationship with other colleagues.” The internal consistency coefficients (α), the McDonald's ω, the CR, and the AVE of the promotive voice were 0.944, 0.945, 0.957, and 0.818, respectively. The internal consistency coefficients (α), the McDonald's ω, the CR, and the AVE of the prohibitive voice were 0.896, 0.897, 0.920, and 0.660, respectively.

#### Creativity

The instrument proposed by Gilson et al. (2012) includes subscales of radical and incremental creativity and was used to measure employee creativity. The subscale of radical creativity contains four items, including “When you think of the ideas you come up with at work, to what extent would you characterize them as being... departures from what is currently done or offered.” The subscale of incremental creativity contains three items, including “When you think of the ideas you come up with at work, to what extent would you characterize them as being... incremental improvements upon existing processes or products.” The internal consistency coefficients (α), the McDonald's ω, the CR, and the AVE of the radical creativity were 0.894, 0.895, 0.926, and 0.759, respectively. The internal consistency coefficients (α), the McDonald's ω, the CR, and the AVE of the incremental creativity were 0.899, 0.901, 0.937, and 0.833, respectively.

#### Contradictory thinking

The subscale of contradictory thinking in the Dialectical Thinking Scale for Chinese People developed by Hou (2004) was used to measure contradictory thinking. This subscale contains four items including: “I often find that things contradict themselves.” The internal consistency coefficient (α), the McDonald's ω, the CR, and the AVE of the scale were 0.824, 0.826, 0.884, and 0.655, respectively.

#### Control variables

According to the recommendations of Chow (2020), this study included in the model the following as control variables: sex, age, marital status, educational background, and years working in the current firm. Specifically, sex (male = 1 and female = 2), marital status (married = 1 and unmarried = 2), and education (senior high school and lower level = 1, junior college = 2, and undergraduate and higher level = 3) were categorical variables, while age and years working at the firm were continuous variables.

## Results

### Quality of the data

The present study used Mplus Version 7.4 to conduct confirmatory factor analysis. The results showed that, among the “leader humor + prohibitive voice + radical creativity + contradictory thinking” models, the four-factor model fit the data better than alternative models (χ^2^ = 489.046, χ^2^*/df* = 4.328, *TLI* = 0.954, *CFI* = 0.945, *SRMR* = 0.040, *RMSEA* = 0.064); among the “leader humor + promotive voice + incremental creativity + contradictory thinking” models, the four-factor model fit the data better than alternative models (χ^2^ = 305.884, χ^2^*/df* = 3.641, *TLI* = 0.969, *CFI* = 0.975, *SRMR* = 0.043, *RMSEA* = 0.057).^1^ To avoid the impact of the standard deviation, Harman's single-factor test was used to examine homology bias through SPSS 22.0. The result found that the first factor explained 38.750% of the total variation, which was lower than the 40% standard. Therefore, the standard deviation of the data was within an acceptable range.

### Correlation analysis

Table 1 shows the data characteristics of each variable, including the mean value, standardized deviation, and the correlation coefficient. The results showed that leader humor was positively correlated with radical creativity (*r* = 0.294, *p* < 0.01) and incremental creativity (*r* = 0.297, *p* < 0.01). Leader humor was also positively correlated with a promotive voice (*r* = 0.295, *p* < 0.01), a prohibitive voice (*r* = 0.302, *p* < 0.01), and contradictory thinking (*r* = 0.117, *p* < 0.01). Moreover, a prohibitive voice was positively correlated with radical creativity (*r* = 0.523, *p* < 0.01), while a promotive voice was positively correlated with incremental creativity (*r* = 0.604, *p* < 0.01). These findings provide a basis for further hypothesis testing.

**Table 1:** Correlation analysis results.

**Variables**	**Means (*SD*)**	**1**	**2**	**3**	**4**	**5**	**6**	**7**	**8**	**9**	**10**	**11**
1 LH	4.47 (1.45)											
2 PROH	4.62 (1.07)	0.295^**^										
3 PROM	4.86 (1.13)	0.302^**^	0.692^**^									
4 CONTR	4.37 (1.18)	0.117^**^	0.111^**^	0.050								
5 OS	1.53 (0.50)	−0.094^**^	−0.162^**^	−0.178^**^	0.105^**^							
6 RC	4.54 (1.05)	0.294^**^	0.523^**^	0.567^**^	0.162^**^	−0.094						
7 IC	4.95 (1.02)	0.297^**^	0.512^**^	0.604^**^	0.090^**^	−0.155^**^	0.634^**^					
8 SEX	1.57 (0.50)	−0.070^**^	−0.183^**^	−0.197^**^	0.037	0.172^**^	−0.139^**^	−0.132^**^				
9 AGR	31.95 (8.06)	−0.064	0.199^**^	0.245^**^	−0.054	−0.075^*^	0.077^*^	0.093^**^	0.002			
10 EDU	2.54 (0.94)	0.096^**^	−0.010	0.078^*^	−0.067	−0.296^**^	0.067	0.115^**^	−0.225^**^	−0.112^**^		
11 MARY	1.62 (0.49)	−0.097^**^	0.127^**^	0.107^**^	−0.025	−0.010	0.044	0.053	0.056	0.569^**^	−0.152^**^	
12 YEAR	5.97 (6.94)	−0.070^**^	0.092^**^	0.148^**^	0.054	0.162^**^	0.099^**^	0.085^*^	−0.035	0.600^**^	−0.124^**^	0.347^**^

(1) ^*^p < 0.05; ^**^p < 0.01 (two-tailed). (2) TS: LH, leader humor; PROH, prohibitive voice; PROM, promotive voice; CONTR, contradiction; OS, ownership; RC, radical creativity; IC, incremental creativity; MAR, marital status.

### Hypothesis testing

An ordinary least squares (OLS) regression was used to test H1 and H2, and the results are shown in Table 2. Leader humor (collected at time point 1) had a significant positive correlation with radical creativity (*adjusted R*^2^ = 0.117, *B* = 0.213, *p* < 0.01) and incremental creativity (*adjusted R*^2^ = 0.123, *B* = 0.208, *p* < 0.01) (collected at time point 3). Hence, H1a and H1b were supported. In addition, leader humor was positively correlated with a promotive voice (*adjusted R*^2^ = 0.194, *B* = 0.236, *p* < 0.01) and a prohibitive voice (*adjusted R*^2^ = 0.167, *B* = 0.223, *p* < 0.01) (collected at time point 2). Next, Model 4 was constructed in PROCESS (Hayes, 2017) to test the mediating effect. The results showed that prohibitive voice mediated the influence of leadership humor on radical creativity (*indirect effect* = 0.105, *SE* = 0.018, 95% CI = 0.071 to 0.141; *direct effect* = 0.109, *SE* = 0.023, 95% CI = 0.064 to 0.153; *total effect* = 0.213, *SE* = 0.024, 95% CI = 0.166 to 0.261, the promotive voice was controlled). Promotive voice mediated the relationship between leader humor and incremental creativity (*indirect effect* = 0.122, *SE* = 0.019, 95% CI = 0.087 to 0.161; *direct effect* = 0.085, *SE* = 0.021, 95% CI = 0.045 to 0.126; *total effect* = 0.208, *SE* = 0.024, 95% CI = 0.162 to 0.254, the prohibitive voice was controlled). Therefore, H2a and H2b were supported.

**Table 2:** Regression analysis results.

**Variables**	**PROH**			**PROM**			**RC**			**IC**		
	***B***	***SE B***	β	***B***	***SE B***	β	***B***	***SE B***	β	***B***	***SE B***	β
LH	0.223	0.024	0.303^**^	0.236	0.025	0.304^**^	0.213	0.024	0.295^**^	0.208	0.023	0.295^**^
SEX	−0.384	0.071	−0.178^**^	−0.379	0.074	−0.167^**^	−0.233	0.072	−0.110^**^	−0.190	0.070	−0.092^**^
AGR	0.027	0.006	0.207^**^	0.037	0.006	0.264^**^	0.003	0.006	0.022	0.008	0.006	0.059
EDU	−0.060	0.038	−0.053	0.050	0.040	0.042	0.038	0.039	0.034	0.095	0.038	0.087^*^
MAY	0.121	0.087	0.055	−0.004	0.090	−0.002	0.084	0.088	0.039	0.094	0.085	0.045
YEAR	−0.007	0.006	−0.043	0.002	0.006	0.011	0.014	0.006	0.093	0.009	0.006	0.062
ADR2	0.167			0.194			0.117			0.123		

(1) ^*^p < 0.05; ^**^p < 0.01. (2) TS: LH, leader humor; PROH, prohibitive voice; PROM, promotive voice; RC, radical creativity; I, incremental creativity; MAR, marital status.

Next, H3 was tested by examining the significance of the interaction item (leader humor × contradiction) (Table 3). The results showed that leader humor was positively correlated with prohibitive voice (*B* = 0.073, *p* < 0.01) and contradictory thinking (*B* = 0.065, *p* < 0.01). Then, the interaction effect was significant (*B* = 0.050, *p* < 0.01), and the model explained more variance (*adjusted R*^2^ = 0.516, Δ*R*^2^ = 0.010, *p* < 0.01). Model 1 was then used in PROCESS (Hayes, 2017) to further test the moderating effect. The results showed that, when contradictory thinking was high, the effect of leader humor on prohibitive voice was significant (*effect* = 0.126, *SE* = 0.023, 95% CI = 0.080 to 0.172), and when contradictory thinking was low, the effect was not significant (*effect* = 0.014, *SE* = 0.024, 95% CI = −0.034 to 0.061). Thus, H3b was supported. Model 7 was used in PROCESS (Hayes, 2017) to test the effect of contradictory thinking on the mediating model. The results revealed that the indirect effect of leader humor on radical creativity was significant when contradictory thinking was high (*indirect effect* = 0.030, *SE* = 0.009, 95% CI = 0.014 to 0.049), the indirect effect was not significant when contradictory thinking was low (indirect effect = 0.003, SE = 0.009, 95% CI = −0.015 to 0.021), and that the indirect effect was significant when contradictory thinking was medium (indirect effect = 0.015, SE = 0.007, 95% CI = 0.003 to 0.029). The moderated mediating effect of the overall model was significant (*index* = 0.012, *SE* = 0.053, 95% CI = 0.003 to 0.024). Hence, H4b was supported. The same methods were used to test H3a and H4a. Leader humor was found to be significantly correlated with promotive voice (*B* = 0.091, *p* < 0.01). However, there was no significant correlation between contradictory thinking and promotive voice (*B* = −0.021, *ns*). In addition, the effect of the interaction term was also not significant (*B* = −0.023, *ns*). Therefore, H3a was rejected (Table 4), and further testing for H4a and H5a was unnecessary.

**Table 3:** The moderating effect of contradictory thinking on the relationship between leader humor and the prohibitive voice.

**Variables**	**PROH**			**PROH**		
	***B***	***SE B***	β	***B***	***SE B***	β
LH	0.073	0.020	0.099^**^	−0.148	0.058	−0.201^*^
PROM	0.607	0.026	0.640^**^	0.604	0.026	0.637^**^
CONTR	0.065	0.023	0.072^**^	−0.163	0.060	−0.181^**^
LH ^*^ CONTR				0.050	0.012	0.428^**^
SEX	−0.160	0.056	−0.074	−0.151	0.056	−0.070^**^
AGR	0.006	0.005	0.048	0.007	0.005	0.054
EDU	−0.085	0.030	−0.074^**^	−0.076	0.029	−0.066^*^
MAY	0.123	0.067	0.056	0.120	0.066	0.055
YEAR	−0.009	0.005	−0.060	−0.010	0.005	−0.065^*^
R^2^	0.506			0.516		
ΔR^2^				0.010^*^		

(1) ^*^p < 0.05; ^**^p < 0.01. (2) TS: LH, leader humor; PROH, prohibitive voice; PROM, promotive voice radical creativity; CONTR, contradictory thinking.

**Table 4:** The moderating effect of contradictory thinking on the relationship between leader humor and the promotive voice.

**Variables**	**PROM**			**PROM**		
	***B***	***SE B***	β	***B***	***SE B***	β
LH	0.091	0.020	0.117^**^	0.193	0.060	0.249^**^
PROH	0.659	0.028	0.625^**^	0.666	0.029	0.632^**^
CONTR	−0.021	0.024	−0.022	0.085	0.064	0.090
LH ^*^ CONTR				−0.023	0.013	−0.190
SEX	−0.124	0.059	−0.055^*^	−0.126	0.058	−0.055^*^
AGR	0.018	0.005	0.131^**^	0.018	0.005	0.127^**^
EDU	0.088	0.031	0.073^**^	0.084	0.031	0.070^**^
MAY	−0.083	0.070	−0.036	−0.083	0.070	−0.036
YEAR	0.007	0.005	0.041	0.007	0.005	0.043
R^2^	0.518			0.520		
ΔR^2^				0.002		

(1) ^*^p < 0.05; ^**^p < 0.01. (2) TS: LH, leader humor; PROH, prohibitive voice; PROM, promotive voice radical creativity; CONTR, contradictory thinking.

Finally, Model 11 was applied in PROCESS (Hayes, 2017) to conduct a three-way interaction analysis. The results showed that the effect of the overall model was not significant (*index* = 0.037, *SE* = 0.010, 95% CI = −0.014 to 0.023). However, in SOEs, the moderated mediation model was significant (*indirect effect* = 0.013, *SE* = 0.007, 95% CI = 0.002 to 0.029). In POEs, the moderated mediation model was not significant (*indirect effect* = 0.009, *SE* = 0.007, 95% CI = −0.004 to 0.025); thus, H5b was partially supported.

**Figure 2:**
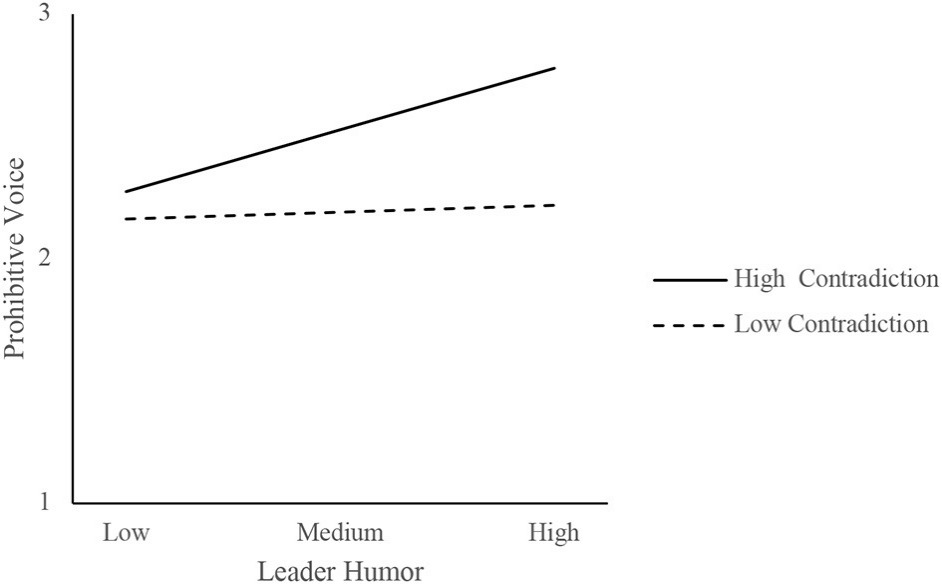
The moderating effect of contradictory thinking on the relationship between leader humor and a prohibitive voice.

**Figure 3:**
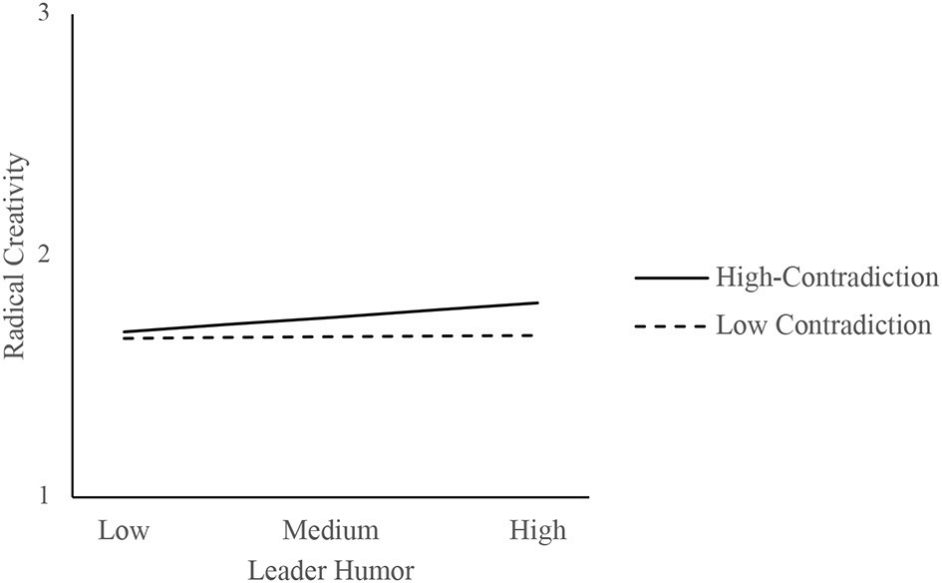
The moderating effect of contradictory thinking on the indirect effect of leader humor on radical creativity.

**Figure 4:**
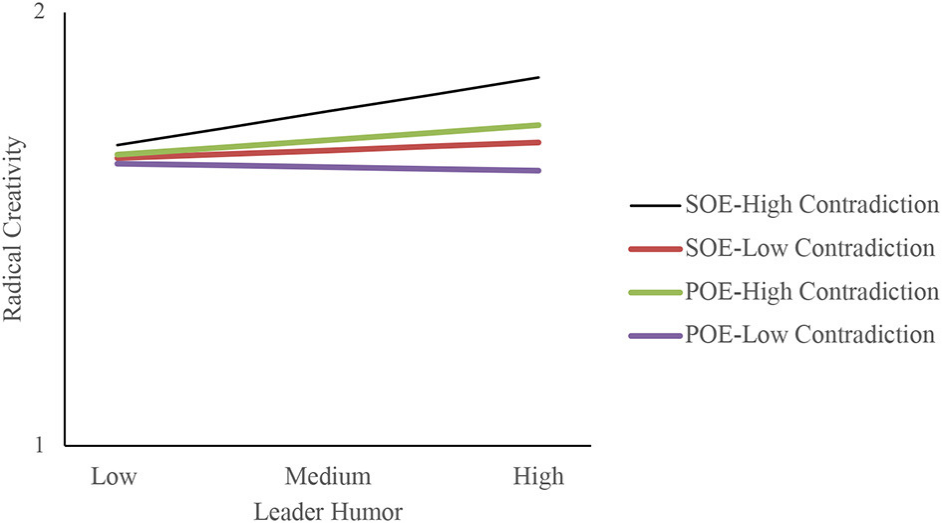
Three-way interaction of leader humor and contradictory thinking and corporate ownership on radical creativity through a prohibitive voice.

Furthermore, the present study used the simple slope method to draw the chart. Low/high levels of contradictory thinking were calculated using –/+ one standard deviation from the mean of the variable. Figure 2 shows that leader humor had a stronger positive influence on prohibitive voice when contradictory thinking was high and a weaker influence when contradictory thinking was low, consistent with H3b. Figure 3 shows that leader humor had a stronger indirect effect on radical creativity when contradictory thinking was high and a weaker indirect effect when contradictory thinking was low. Figure 4 shows that, among the four scenarios (“SOE-high contradiction,” “SOE-low contradiction,” “POE-high contradiction,” and “POE-low contradiction”), leader humor had a stronger indirect effect on radical creativity in the “SOE-high contradiction” case.

## Discussion

### Theoretical contributions

This study makes the following four theoretical contributions. First, the results showed that leader humor had a significant positive effect on employee incremental and radical creativity. Previous studies have conceptualized and operationalized creativity as a single concept, which overlooks its inherent complexity. This study further divided creativity into radical and incremental creativity according to the recommendations of Madjar et al. (2011). In this respect, these findings not only contribute to the literature on leadership but also enrich the existing theories of creativity. Second, based on the BVT, the results showed that leader humor has a positive impact on both promotive and prohibitive types of voice. This result is consistent with the conclusions of previous research (Lin, 2016; Tan et al., 2021). More importantly, this study presents the first efforts to distinguish the mechanisms by which leader humor influences different types of creativity. In specific, the results showed that a promotive voice mediates the impact of leader humor on incremental creativity, whereas a prohibitive voice mediates the association between leader humor and radical creativity. Thus, different types of employee voices may lead to different types of creativity.

Third, this study found that contradictory thinking moderates the effect of leader humor on employee voice. Compared to employees with low levels of contradictory thinking, employees with high levels of contradictory thinking are more likely to perceive leader humor and thus express their voice. However, it was also found that contradictory thinking only moderates the relationship between leader humor and prohibitive voice but not between leader humor and promotive voice. This may be because Chinese culture emphasizes maintaining and prioritizing harmony. Thus, compared to the prohibitive voice that may put employees' relationships with leaders at risk, Chinese employees are more inclined to propose suggestions that maintain internal unity and stability, such as a promotive voice. Consequently, such social desirability for a promotive voice may make the participants exhibit bias in the process of completing the questionnaire.

Fourth, the moderated mediation effect was supported in SOEs but not in POEs. This conclusion was contrary to our hypothesis. The result further found that the indirect effect of leader humor on the radical creativity of employees with high levels of contradictory thinking through prohibitive voice is greater in SOEs than in POEs (“SOE-high contradiction” > “POE-high contradiction”). We attributed this phenomenon to the following reasons: There has been a growing demand for employment in China over recent years, which may result in increased difficulty in finding a new job. As a result, employees may be less willing to risk losing their current job to exhibit a prohibitive voice. Compared to employees in POEs, employees in SOEs are better protected (Sheng and Zhao, 2013) by law and thus have the courage to exhibit a prohibitive voice. However, employees in POEs have lower job security, and even if they perceive leader humor, they are unlikely to exhibit a prohibitive voice that may challenge their relationships with the leaders. Moreover, compared to POEs, SOEs are more likely to obtain external resources from universities, scientific institutions, and the government (Liu et al., 2017) to facilitate innovation in China (Choi et al., 2011). In contrast, POEs have fewer resources and are thus less capable of implementing innovation changes. Even though leaders are well aware of the prohibitive voice, the implementation of such suggestions is likely restricted due to limited resources in POEs. Therefore, employees in POEs may not have a stronger motivation to exhibit a prohibitive voice.

### Practical implications

This study has some practical implications. First, this study found that radical and incremental creativity are not mutually exclusive, and thus, we suggest that managers should regard humor as an interpersonal resource that enhances employee creativity. Second, the findings showed that leader humor stimulates employee creativity by letting them voice their opinions. Therefore, we recommend that enterprises should provide communication training to managers and further encourage them to communicate with employees humorously. Third, we recommend that POEs adopt some management measures to enhance employees' job security, such as offering stock ownership plans or long-term work contracts. In addition, to encourage innovative projects in POEs, we recommend that the government provide them with more resources and opportunities for trial and error.

### Limitations and future research

This study has some limitations that should be considered. First, social desirability may have influenced the participants' evaluation of the questionnaire on the concept of a promotive voice. Future research should control for such an effect. Second, all variables in this study were self-reported by participants. Although the standard deviation of the data was within an acceptable range, this design inevitably led to some bias. We recommend that multisource data collection be used in the future, such as collecting another set of data with multiple sources of data (leaders and followers) and attempting to replicate the outcomes. Third, we used questionnaires to explore how leader humor influences two types of creativity, but this method may not provide evidence for causal relations between variables. Future studies should investigate causality through an experimental design.

## Data availability statement

The raw data supporting the conclusions of this article will be made available by the authors, without undue reservation.

## Ethics statement

The studies involving human participants were reviewed and approved by the Peking University. The patients/participants provided their written informed consent to participate in this study.

## Author contributions

YC and KZ developed the study concept, drafted the manuscript, and were performed the testing and data collection. YH and RM contributed to the study design and provided critical revisions. YC and YW performed the data analysis and interpretation under the supervision of YH and RM. All authors approved the final version of the manuscript for submission.

## Footnotes

^1^The Composite Reliability (CR) and the Average Variance Extracted (AVE) of all factors are greater than 0.7 and 0.5 respectively.
